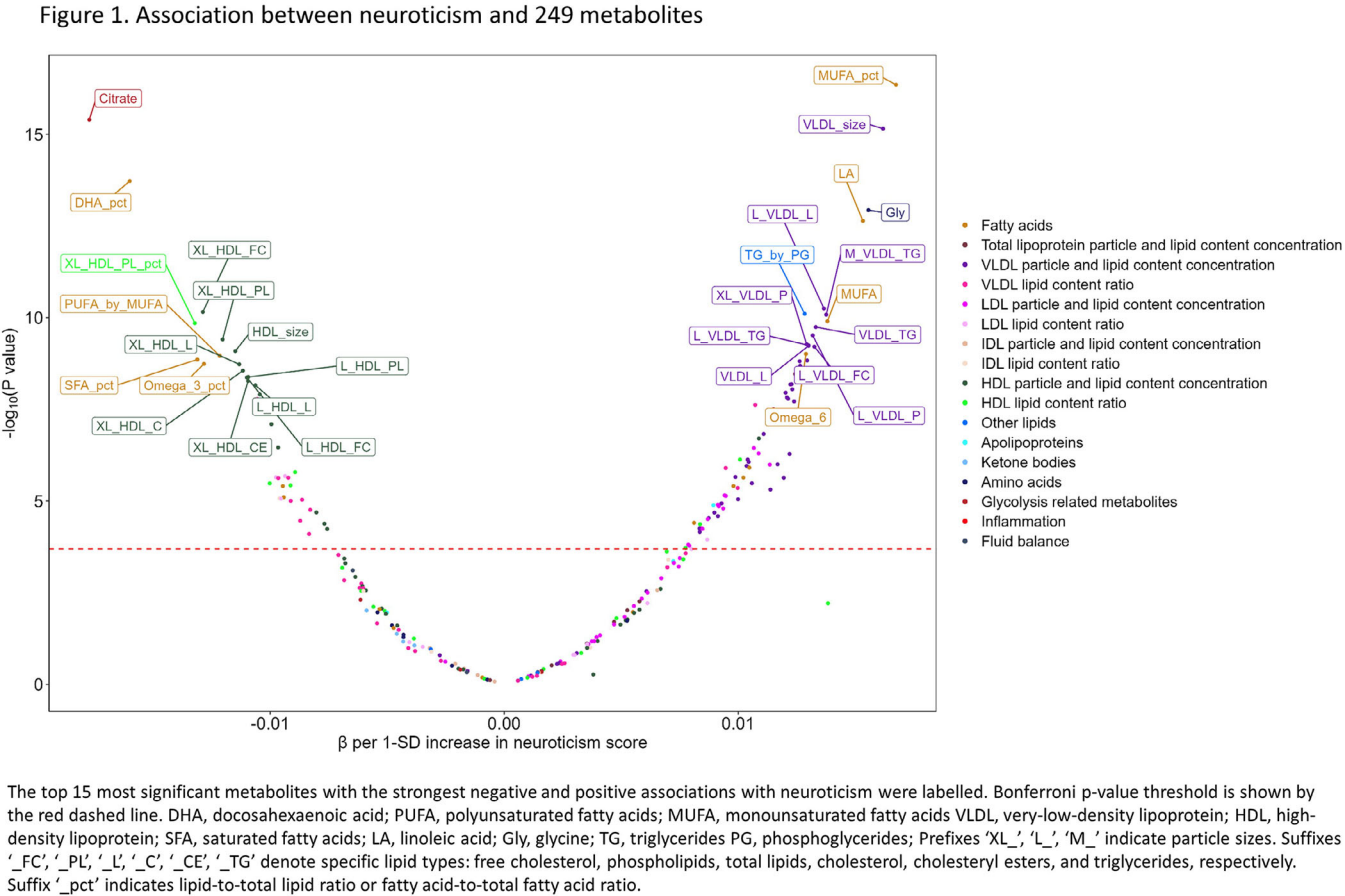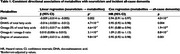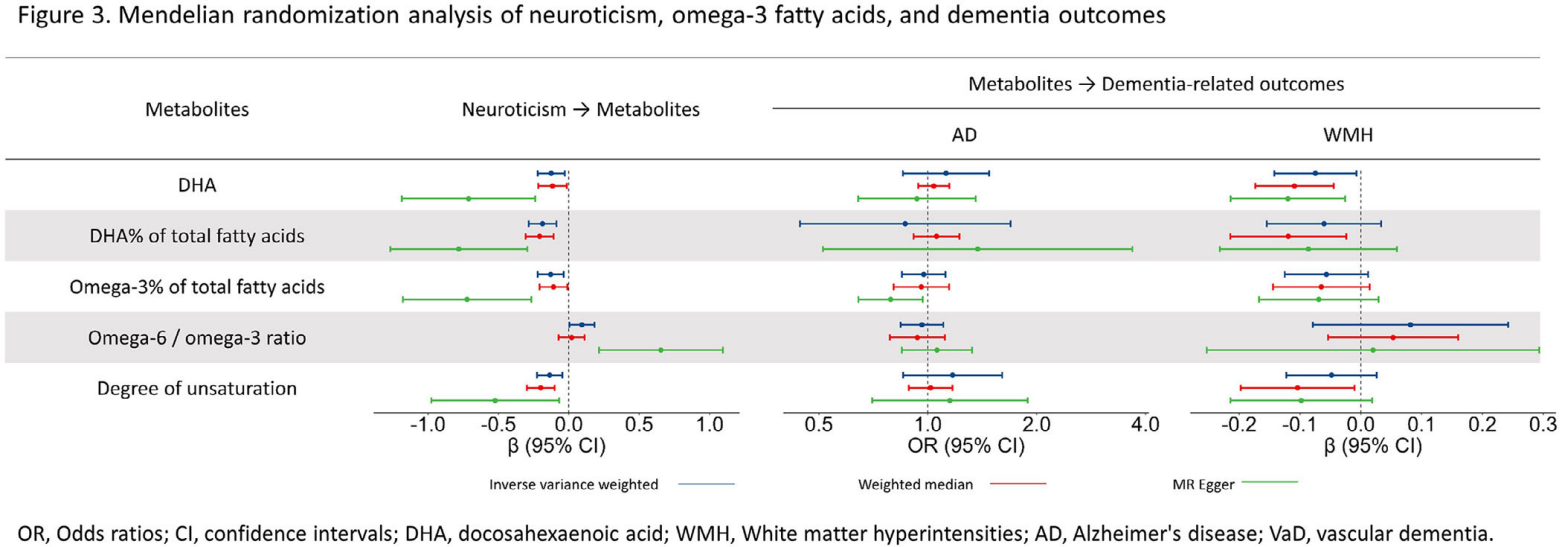# Neuroticism, omega‐3 fatty acids, and risk of incident dementia

**DOI:** 10.1002/alz70860_104686

**Published:** 2025-12-23

**Authors:** Yaqing Gao, Cornelia M Van Duijn, Thomas J Littlejohns, Najaf Amin

**Affiliations:** ^1^ University of Oxford, Oxford, Oxfordshire, United Kingdom

## Abstract

**Background:**

High levels of neuroticism are associated with an increased risk of dementia, yet the underlying biological mechanisms remain poorly understood. Investigating the role of metabolites, the downstream products of metabolic processes, may offer valuable insights into this association.

**Method:**

In 215,624 dementia‐free UK Biobank participants aged 40–69 years, we used linear regression to examine cross‐sectional associations between neuroticism and 249 metabolites quantified by nuclear magnetic resonance spectroscopy (Nightingale Health Plc), adjusting for sex, age, assessment centre, spectrometer, ethnicity, socioeconomic deprivation, education, smoking status, alcohol consumption, body mass index, and common medications that may affect metabolic processes (e.g., lipid‐lowering drugs). Metabolites reaching Bonferroni‐corrected significance were further tested for associations with incident all‐cause dementia, Alzheimer's disease (AD) and vascular dementia (VaD) using Cox proportional‐hazards regression, and with white matter hyperintensities volume using linear regression. Causality in significant observational relationships was evaluated through two‐sample Mendelian randomization.

**Result:**

Neuroticism was significantly associated with 119 out of 249 metabolites (Bonferroni‐adjusted *p* <0.05). Among these, five metabolites involved in fatty acid metabolism showed consistent directional associations with both neuroticism and incident all‐cause dementia. Specifically, four metabolites, including docosahexaenoic acid (DHA), DHA% of total fatty acids, omega‐3% of total fatty acids, and degree of unsaturation, were associated with lower neuroticism levels and a decreased risk of incident dementia. Conversely, the omega‐6/omega‐3 ratio was positively associated with both neuroticism and dementia risk. Associations between these five metabolites and VaD were stronger than those with AD. Mendelian randomization analysis suggested that high levels of neuroticism reduce DHA levels, which, in turn, contribute to white matter pathology, a hallmark of VaD.

**Conclusion:**

Neuroticism is associated with lower levels of omega‐3 fatty acids, particularly DHA, which may increase dementia risk, primarily through cerebrovascular mechanisms. Further research with temporally sequenced measures of neuroticism, metabolites, and dementia is needed to strengthen evidence for this pathway.